# Prevention of Physicians’ Suicide

**Published:** 2014

**Authors:** Fatemeh Sheikhmoonesi, Mehran Zarghami

**Affiliations:** 1 Associate Professor, Psychiatry and Behavioral Sciences Research Center, Addiction Institute AND Department of Psychiatry, Mazandaran University of Medical Sciences, Sari, Iran.; 2 Professor, Psychiatry and Behavioral Sciences Research Center, Addiction Institute AND Department of Psychiatry, Mazandaran University of Medical Sciences, Sari, Iran.

## Abstract

Suicide rate in physicians has been reported to be higher than general population or other academics. Previous studies found that 85-90% of people who commit suicide had been suffering from some type of psychiatric disorder. Suicide prevention is the key element in lowering the numbers of physicians who destroy themselves and end their lives each year. It is needed to provide some educational programs to increase physicians’ awareness of warning signs of suicidal ideation such as observable signs of serious depression. According to previous studies, some specialties such as psychiatry, anesthesiology, and dentistry are at higher risk for physicians’ suicide. Hence, it is important to select candidates in these fields carefully as a primary prevention program.

World Health Organization (WHO) defined suicide as “an act with a fatal outcome, which the deceased knowing or expecting a fatal outcome had initiated and carried out with the propose of provoking the change that he desired” (1). Suicide rate in physicians has been reported to be higher than general population or other academics in many studies (2, 3). For example, Frank et al. in a national study on causes of death found that mortality due to suicide and self-injury among white male USA physicians is 70% higher than other professionals. They have reported that suicide rate among female physicians is 3-4 times more prevalent than the general population (4).

Suicide rates increase abruptly by age among doctors (5). Previous studies found that 85-90% of people who commit suicide had been suffering from some type of psychiatric disorder (6). Frank and Dingle reported the history of depression, cigarette smoking, alcohol abuse or dependence, sexual abuse, domestic violence, poor current mental health, more sever harassment, and family history of psychiatric disorder as some characteristics of women physicians who had attempted suicide (7). Furthermore, orphaned and childless physicians, and those who have home stressors, obesity, chronic fatigue syndrome, worse health, eating disorders, substance abuse, hardworking, career displeasure, and job stressors have been reported as high risk physicians (7). On the other hand, some other studies indicate that drug abuse, alcoholism, marital discord, and admission to psychiatric institutions are prevalent among the medical profession (3).

Gold et al. found that all medical specialties are not equally influenced by substance dependence and abuse. Anesthesiologists are more engaged with drug abuse disorders significantly (8). Easy access to drugs with high abuse potential may be an important factor in physician suicide attempts.

Epstein et al. reported some psychological characteristics, which have predictive value in identifying future physicians’ suicide. Many personality factors, particularly self-destructive tendency, depression, and guilty self-concept are significantly higher among suicidal physicians than the controls (9). Gagne et al. found that borderline personality disorder is more prevalent among physicians who committed suicide (10).

Physicians’ suicide has also been associated with marital status, relationship and occupational difficulties, and financial problems as well as alcoholism, drug abuse, and positive psychiatric history (10).

The literature also suggests that physicians who kill themselves are more critical of others and of themselves and more likely to blame themselves for their own illnesses. Furthermore, there is some evidence that physicians do not welcome to colleagues for help, and instead utilize alcohol or drugs and resort to isolation. Besides, once they look for help it seems that they are not taken seriously and adequately by their colleagues (2).

Previous researchers found that more than 50% of physicians who sought help and later committed suicide had been diagnosed with psychiatric disorders (11); most of them were not hospitalized before death (12).

Several conceptual models have been put forward to prevent this complex phenomenon. Suicide prevention is the key element in lowering the numbers of physicians who destroy themselves and end their lives each year. Preven in a primary prevention movement suggested that medical school curriculum should include programs to advance more self-awareness in students of their emotional needs. Furthermore as a secondary prevention, any intervention requires that doctors have the ability to believe that anyone, regardless of status, may be suicidal. Besides, professional roles should not limit coworkers and friends from identifying preliminary signals. Finally, tertiary prevention offers the survivors the chance to deal with the searing loss in a therapeutic manner (13).

The first suicide prevention and depression awareness program was established at the University of California in 2009 to destigmatize help-seeking and prevent suicide among medical student, residents and faculty physicians. The approach of the program consisted of screening, assessment, referral and education. The screening practice was trustworthy. The educational part consisted of Grand Rounds on students’ and physicians’ exhaustion, depression, and suicide (14).

From ecological point of view, two main components of suicide prevention are identification of vulnerable groups and individuals, and limitation of access to specific methods of suicide (15).

Unfortunately, there is no documented data on prevalence of physicians’ suicide and related factors in Iran. It seems that medical professional suicides can be categorized in two major classes: 1) The young student especially girls that face to emotional conflict. 2) The hard working residents and specialists. However, it is recommended to screen suicide risk factors such as medical (depression, drug abuse, borderline personality disorder, and other psychiatric disorders), and sociological factors (particularly anomic factors as a result of weak social protective elements) in medical students, as well as residents, while they enter to medical university and when they want to choose their specialty field. According to previous studies some specialties such as psychiatry, anesthesiology, and dentistry are at higher risk for physicians’ suicide (16). So it is important to select candidates in these fields carefully as a primary prevention program. More severe regulations and limitation of access to the sedative, hypnotic, analgesic as well as stimulant drugs, which have abuse potential and consequently addiction and vulnerability to suicide is another effective needed primary preventive attempt. Also, we should keep in mind that when vulnerable individuals face an important event or conflict beyond their capacity to solve problems, they tend to resort to suicide (17). Counseling and psychotherapeutic assistance for medical students and physicians who are stressed and burned-out can implement another effective preventive program. It is needed to provide some educational programs to increase physicians’ awareness of warning signs of suicidal ideation such as observable signs of serious depression. As Preven noted long ago, “If the physician cannot heal himself, perhaps he can learn to recognize the need for assistance” (13).

## Footenote

**Declaration of interest:** None.

*This article has its roots in my consternation over the loss of my medical school classmate: Dr. “N.F.”*

*(F. Sheikhmoonesi)*
